# Development and Optimization of Chitosan Nanoparticle-Based Intranasal Vaccine Carrier

**DOI:** 10.3390/molecules27010204

**Published:** 2021-12-29

**Authors:** Xiaoyi Gao, Nan Liu, Zengming Wang, Jing Gao, Hui Zhang, Meng Li, Yimeng Du, Xiang Gao, Aiping Zheng

**Affiliations:** State Key Laboratory of Toxicology and Medical Countermeasures, Beijing Institute of Pharmacology and Toxicology, Beijing 100850, China; gaoxiaoyiemail@163.com (X.G.); wowlinan@sohu.com (N.L.); wangzm.1986@163.com (Z.W.); gjsmmu@126.com (J.G.); zhhui58@126.com (H.Z.); limeng854321@126.com (M.L.)

**Keywords:** chitosan nanoparticle, vaccine carriers, intranasal administrated vaccine/intranasal administration/intranasal vaccine, design of experiment, D-mannose

## Abstract

Chitosan is a natural polysaccharide, mainly derived from the shell of marine organisms. At present, chitosan has been widely used in the field of biomedicine due to its special characteristics of low toxicity, biocompatibility, biodegradation and low immunogenicity. Chitosan nanoparticles can be easily prepared. Chitosan nanoparticles with positive charge can enhance the adhesion of antigens in nasal mucosa and promote its absorption, which is expected to be used for intranasal vaccine delivery. In this study, we prepared chitosan nanoparticles by a gelation method, and modified the chitosan nanoparticles with mannose by hybridization. Bovine serum albumin (BSA) was used as the model antigen for development of an intranasal vaccine. The preparation technology of the chitosan nanoparticle-based intranasal vaccine delivery system was optimized by design of experiment (DoE). The DoE results showed that mannose-modified chitosan nanoparticles (Man-BSA-CS-NPs) had high modification tolerance and the mean particle size and the surface charge with optimized Man-BSA-CS-NPs were 156 nm and +33.5 mV. FTIR and DSC results confirmed the presence of Man in Man-BSA-CS-NPs. The BSA released from Man-BSA-CS-NPs had no irreversible aggregation or degradation. In addition, the analysis of fluorescence spectroscopy of BSA confirmed an appropriate binding constant between CS and BSA in this study, which could improve the stability of BSA. The cell study in vitro demonstrated the low toxicity and biocompatibility of Man-BSA-CS-NPs. Confocal results showed that the Man-modified BSA-FITC-CS-NPs promote the endocytosis and internalization of BSA-FITC in DC2.4 cells. In vivo studies of mice, Man-BSA-CS-NPs intranasally immunized showed a significantly improvement of BSA-specific serum IgG response and the highest level of BSA-specific IgA expression in nasal lavage fluid. Overall, our study provides a promising method to modify BSA-loaded CS-NPs with mannose, which is worthy of further study.

## 1. Introduction

Currently, a vaccine seems to be the most effective strategy to manage the pandemic of COVID-19 [1,2]. Up to now, more than 160 types of COVID-19 vaccine are undergoing preclinical or clinical trials [3]. Unfortunately, a systemic vaccine was reported to be not able to prevent SARS-CoV-2 nasal infection and asymptomatic transmission [3]. A study published in *Nature* reported that “protection in both the upper and lower respiratory tracts will be required to prevent transmission and disease in humans” [4]. Wei Chen, an academician at the Chinese Academy of Engineering, indicated that an intranasal vaccine was more effective in creating mucosal immunity against SARS-CoV-2. Chen’s group have developed an aerosolised adenovirus type-5 vector-based COVID-19 vaccine (Ad5-nCoV) [5] and evaluated its safety and immunogenicity in adults [6], which is well tolerated and effective in phase I clinical trials [6].

Compared with a systemic vaccine, an intranasal vaccine can effectively stimulate the protective immune response of upper respiratory tract mucosa and distal mucosal sites such as lung mucosa, gastric mucosa and reproductive tract mucosa [7,8]. Due to the large absorption area, rich blood flow and thin endothelial basement membrane of the nasal cavity, an intranasal vaccine can achieve the desired immune effect at a lower dose than a systematic vaccine [5]. However, the development of an intranasal vaccine has suffered from limitations including the rapid ciliary clearance of nasal mucosa, enzymatic degradation, and the low bioavailability of vaccines [9]. The nasal cilia keep clearing mucus and substances (pathogens, particles, and xenobiotics) that adhere to the mucosa and, thus, the intranasal vaccine will be cleared quickly [10,11]. Furthermore, because of the higher susceptibility of biological drugs to enzymatic degradation and their low permeability across the epithelium, the absorption of biomacromolecules from nasal mucosa is poor [12]. Therefore, it is necessary to develop vaccine carriers to enhance the bioavailability of the intranasal vaccine.

Chitosan (CS), a polysaccharide acquired from the shells of marine creatures, is a non-toxic, biocompatible, and biodegradable material [13] with mucoadhesion, permeation enhancement as well as efflux pump inhibition [14]. CS nanoparticles (CS-NPs) have attracted much research interest due to their fascinating characteristics. Firstly, under physiological conditions, the positively charged CS-NPs are apt to stick to the negatively charged mucinous protein, which greatly slows down the ciliary clearance of CS-NPs [15]. Secondly, CS can open the tight junction of nasal mucosal epithelial cells, thereby promoting the permeability of CS-NPs [16]. CS-NPs have been proved to be effective carriers for intranasal delivery of biomacromolecules [17]. Moreover, CS-NPs have been reported to improve the potency of vaccines [18,19]. El-Sissi et al. [18] found that CS-NPs enhanced the potency of inactivated Rift Valley Fever Virus vaccine, which is superior to alum in inducing cell-mediated immune response. Vila et al. [19] prepared tetanus toxoid (TT) loaded CS-NPs as an intranasal vaccine. Compared with the intranasally administrated TT solution, TT-loaded CS-NPs produced stronger and more persistent humoral immune response, and more intense mucosal immune response.

There are various methods to prepare CS-NPs [20,21,22], among which the ionic gelation method requires mild conditions without toxic solvents [23]. Tripolyphosphate (TPP), a non-toxic pentavalent crosslinking agent, is the most widely used cross-linker for CS. In the ionic gelation method, CS-NPs are formed instantaneously through the ionic interaction between TPP and CS [13]. The biomacromolecules dispersed in CS or TPP solutions prior to mixing can be loaded into the CS-NPs. The properties of CS-NPs such as size, zeta potential, and loading efficiency can be readily tuned by adjusting preparation parameters. 

Vaccine delivery platforms based on CS and its derivatives have been proven to effectively deliver biomacromolecule, viral or bacterial vaccines to mucosal sites. Various modifications have been applied to improve the properties of CS-based particle systems. Chemical modification of CS to include thiol groups can impart gluing properties, which provide a prolonged residence time by covalently adhering to the mucosal surface [24]. Zhang et al. [25] prepared PEG-g-CS-NPs to facilitate the nasal absorption of insulin. Amidi et al. [26] developed N-trimethyl CS (TMC) NPs loaded with influenza subunit antigen for intranasal vaccination, which had good immunogenicity in a mouse model. It has been reported [27] that the PEGylated CS microspheres loaded with *Bordetella bronchiseptica* dermonecrotoxin (BBD) antigens were physically more stable and stimulated more cytokines from macrophage cells in vitro as compared to BBD-loaded CS microspheres.

In this study, mannose (Man), an FDA-approved pharmaceutical ingredients, was used to modify CS-NPs. Mannose receptor (MR) is mainly expressed by the dendritic cells (DCs) and macrophages [28]. The specific ligand–receptor recognition between MRs and Man can significantly enhance the receptor-mediated endocytosis of Man-modified NPs in DCs and macrophages [29]. In recent years, Man-modified CS-NPs have attracted increasing research attention [29,30]. However, currently, Man is mainly covalently linked to the free amino group of CS by a reductive amination reaction [31], which introduced organic reagents and highly toxic catalysts [30,32,33]. However, in this study, Man-modified CS-NPs were synthesized by a facile ionic gelation method without organic reagents or highly toxic catalysts.

In this study, by utilizing bovine serum albumin (BSA) as a model antigen, intranasal vaccines based on Man modified CS-NPs were developed. A design of experiment (DoE) was used to optimize the synthesis parameters of Man modified CS-NPs loaded with BSA (Man-BSA-CS-NPs). Characterizations of the NPs were performed by dynamic light scattering (DLS), transmission electron microscope (TEM), Fourier transform infrared spectroscopy (FTIR), differential scanning calorimetry (DSC) and sodium dodecyl sulfate polyacrylamide gel electrophoresis (SDS-PAGE) analysis. Furthermore, the interaction between BSA and CS were further investigated by fluorescence spectroscopy analysis. Cytotoxicity, endocytosis and antibody testing of Man-BSA-CS-NPs were also conducted.

## 2. Results and Discussion

### 2.1. Preparation and Characteristics of CS-NPs

**Figure 1:**
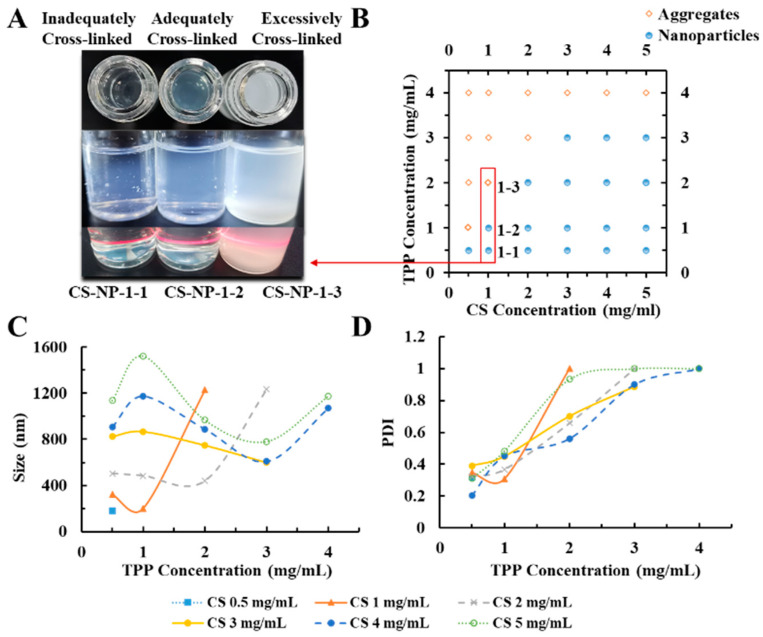
(**A**) The appearances of the chitosan nanoparticles (CS-NPs) in different conditions (Inadequately/Adequately/Excessively Cross-linked); (**B**) effect of tripolyphosphate (TPP) and CS doses on the form (Aggregates/Nanoparticles) of products; (**C**) effect of TPP and CS doses on particle size of CS-NPs; (**D**) effect of TPP and CS doses on PDI of CS-NPs. (CS solution was kept at 5 mL with pH 4.0; the volume ratio of CS solution: TPP solution was kept at 5:2 for all the preparation experiments. TPP solution was slowly added into CS solution under magnetic stirring (15,000 rpm, 30 min) at room temperature).

**Figure 2:**
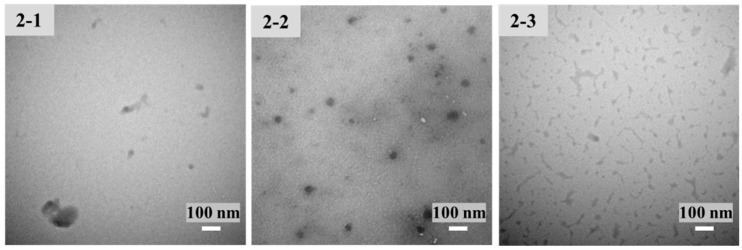
Transmission electron microscopy (TEM) images (at room temperature) of CS-NP-2-1, 2-2 and 2-3 in Table 1.

Under acidic conditions, CS was protonated by the amino group. Due to the opposite charges, CS and TPP were cross-linked by electrostatic interaction. Condition screening of CS-NPs is shown in Figure 1, in which the 1 mg/mL CS solution is most suitable for the preparation of CS-NPs. When TPP was insufficient, the deficient crosslinking of CS led to a large-diameter product with a loose structure. When too much TPP was added, the excessive cross-linking led to agglomerate precipitate. CS-NPs only formed under proper CS:TPP ratio. As shown in Table 1, keeping the concentration of CS at 1 mg/mL, the samples with CS/TPP ratios (*w*/*w*) of 2.2–2.5 were investigated. Although the DLS results of CS-NP-2-1 and CS-NP-2-2 and CS-NP-2-3 had no significant difference, the morphology of the resulting CS-NPs was quite different (Figure 2). Due to the DLS results and the TEM observation, CS-NP-2-2 appeared to be the optimal CS-NPs with minimum particle size, smallest PDI, and spherical morphology.

### 2.2. Preparation and Optimization of Man-BSA-CS-NPs

**Figure 3:**
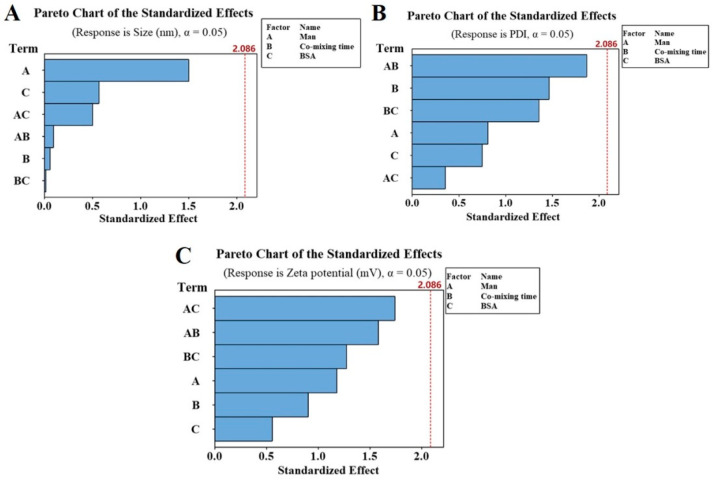
Pareto chart of the standardized effects toward particle size (**A**), polydispersity index (PDI) (**B**) and zeta potential (**C**) of Man-BSA-CS-NPs.

The Man concentration and the mixing time of Man and CS solution are significant factors to introduce Man into CS-NPs. However, the standardized Pareto diagram (Figure 3) showed that none of these variables have significant influence on the particle size, PDI or zeta potential of Man-BSA-CS-NPs within the investigated range. Therefore, the adjustment range of Man, co-mixing time and BSA was large, which provided error tolerance in the following research. 

**Figure 4:**
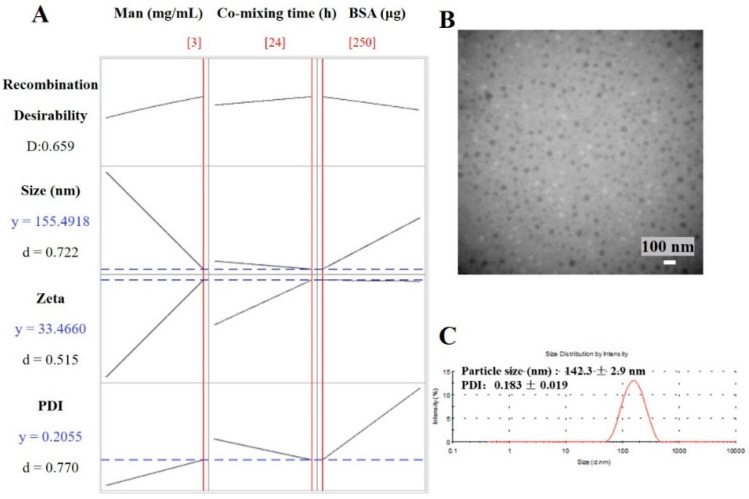
(**A**) Calculated optimization plot (red data/red current line) using the Minitab 10 software profile optimizer tool for Man-BSA-CS-NPs; (**B**) TEM images of optimized Man-BSA-CS-NPs (scale bar: 100 nm); (**C**) DLS image of optimized Man-BSA-CS-NPs. (Preparation condition of optimized Man-BSA-CS-NPs: 3 mg/mL Man, 24 h co-mixing time and 250 μg BSA; the concentration of CS solution was 1 mg/mL; CS solution was kept at 5 mL with pH 4.0; the volume ratio of CS solution: TPP solution was kept at 5:2).

The optimal results from response optimizer in Minitab are shown in Table 2 and Figure 4A. The mathematical model of 3 mg/mL Man, 24 h co-mixing time and 250 μg BSA minimizes the particle size to 156 nm, minimizes the PDI to 0.206 and achieves a 33.5 mV zeta potential. The theoretical values of responses were close to the experimental results. As shown in Figure 4B, the optimized Man-BSA-CS-NPs exhibited uniform spherical morphology. The particle size observed by TEM (about 40 nm) were smaller than that detected (142 ± 3 nm) by DLS, which should be related to different mechanisms of making measurements. DLS measures the hydrodynamic diameter of an equivalent sphere of the NPs. DLS results include CS-NPs and a sphere of hydration around the NPs, compared to typically just NPs being reported by traditional TEM size measurements [34]. In addition, TEM micrographs confirmed the nanosize of dried CS-NPs, and the third dimensional aspects may cause a smaller diameter of CS-NPs. It has also been previously reported [35] that the dried cross-linked NPs (60–280 nm) observed by TEM have a smaller size and a narrower size distribution than the swollen NPs (270–370 nm) detected by DLS.

### 2.3. Characterization of Man-BSA-CS-NPs

#### 2.3.1. Fourier Transform Infrared (FTIR) Characterization

**Figure 5:**
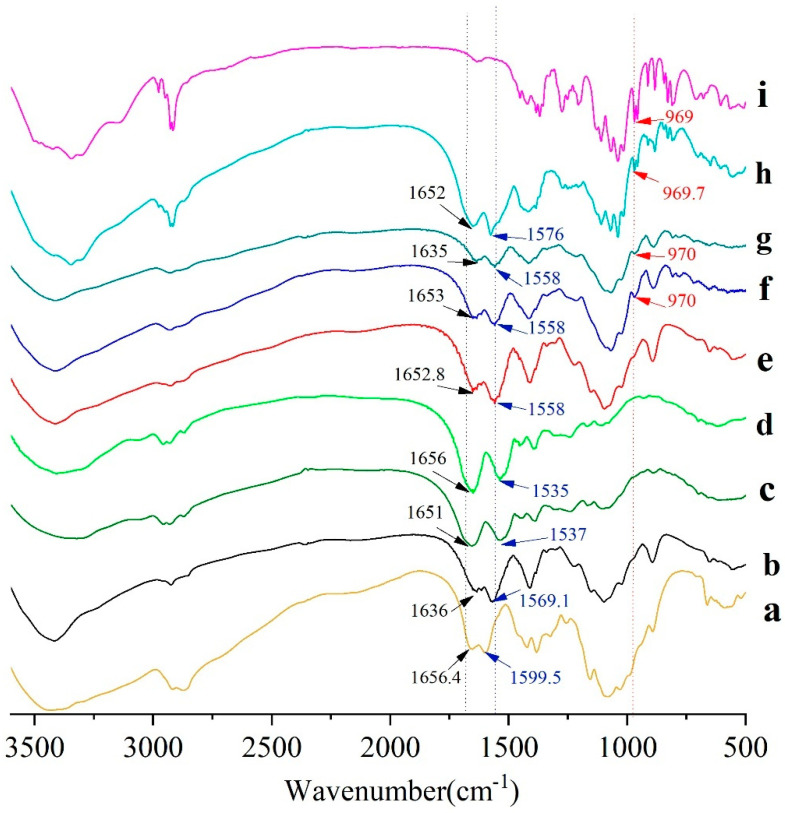
Fourier transform infrared (FTIR) spectra of (**a**) CS; (**b**) CS-NPs; (**c**) BSA; (**d**) a mixture of CS-NPs and BSA; (**e**) BSA-CS-NPs; (**f**) Man-BSA-CS-NPs; (**g**) Man-CS-NPs; (**h**) a mixture of CS-NPs, Man and BSA; (**i**) Man.

FTIR spectra of CS-NPs, BSA, Man, BSA-CS-NPs, Man-CS-NPs, and Man-BSA-CS-NPs are presented in Figure 5. As shown in Figure 5b, unprocessed CS exhibited an intense and broad band at 3429 cm^−1^ attributed to intermolecular hydrogen bond stretching (stretching vibration of -OH groups and N-H) [36] and absorption bands at 2916 cm^−1^, 2873 cm^−1^, 1423 cm^−1^ and 1325 cm^−1^ due to the CH_2_ stretching vibration; C=O in the amide group (amide I band, in incompletely deacetylated CS) appears at 1656 cm^−1^; the absorption band at 1599 cm^−1^ due to NH_2_ in the amino group [37]; the amide III band at 1383 cm^−1^ due to C-N stretching vibration; C-N stretching vibration of amino groups at 1324 cm^−1^ [38]; polysaccharide glycosidic characteristic bands between 893–1156 cm^−1^ (-C-O-C- in glycosidic linkage at 1084–1156 cm^−1^) [37,38], and C-O stretching vibration of primary alcohols at 1084 cm^−1^ [39].

Compared with unprocessed CS, the main changes in the FTIR spectra of the CS-NPs were the absence of bands at 1590 cm^−1^ (corresponding to NH_2_ in amino group) and 1324 cm^−1^ (C–N stretching vibration of amino groups), while a brand at 1569 cm^−1^ (related to ^+^NH_3_) was recorded [36]. These differences were mainly caused by complexation among CS and TPP molecules [39], which is attributed to the linkage between the tripolyphosphate groups of TPP and the ammonium ion of CS. The bands at 1226 cm^−1^ (P=O stretching vibration) and 892 cm^−1^(P–O–P asymmetric stretching) indicate the presence of phosphate groups in the CS-NPs. In addition, the subtle spectral differences in the range from 2000 to 4000 cm^−1^ were assigned to the diversities in the H-bonds intensities among CS–TPP and TPP–TPP chain segments [39].

Unprocessed BSA powder (Figure 5c) has characteristic bands at 3409 cm^−1^, 1651 cm^−1^ (amide I, C=O stretching), 1537 cm^−1^ (amide II, -NH bending), and 1393cm^−1^ (amide III, C–N stretching). The mixture of CS-NP and BSA (Figure 5d) has a similar FTIR spectrum to BSA on the whole, while the FTIR spectrum of BSA-CS-NPs has more in common with CS-NPs. Compared with CS-NPs, the bands of BSA-CS-NPs (Figure 5e) have shifts from 1636 to 1653 cm^−1^, from 1569 to 1558 cm^−1^, which can be ascribed to the electrostatic interaction between the polysaccharide cation of CS and the polyanion of BSA. Therefore, the analysis of the FTIR spectra of the samples confirmed the presence of BSA on the BSA-CS-NPs in contrast with CS-NPs and the mixture of CS-NPs and BSA [40]. 

Pure Man showed characteristic bands (Figure 5i) at 3345.8 cm^−1^ (–OH stretching), 2917.4 cm^−1^ (–CH stretching), 1110 cm^−1^ (C–O stretching, splits into several peaks), 969 cm^−1^ (C–H bending of end-group carbon). In Man-CS-NPs, the characteristic brand of Man at 970 cm^−1^ (C–H bending of end-group carbon) is shown, which also appears in the FTIR spectrum of Man-BSA-CS-NPs. It indicates the presence of Man on Man-CS-NPs and Man-BSA-CS-NPs. In Man-BSA-CS-NPs, the bands of -NH_2_ stretching, amide I and amide II are more rugged than those in Man-CS-NPs, and the peak of amide I (C=O stretching) shifts from 1635 cm^−1^ to 1652.9 cm^−1^. These changes may indicate an increase in the electron absorption effect and ring tension, as well as a decrease in the conjugation effect and hydrogen bonding.

#### 2.3.2. DSC Characterization

**Figure 6:**
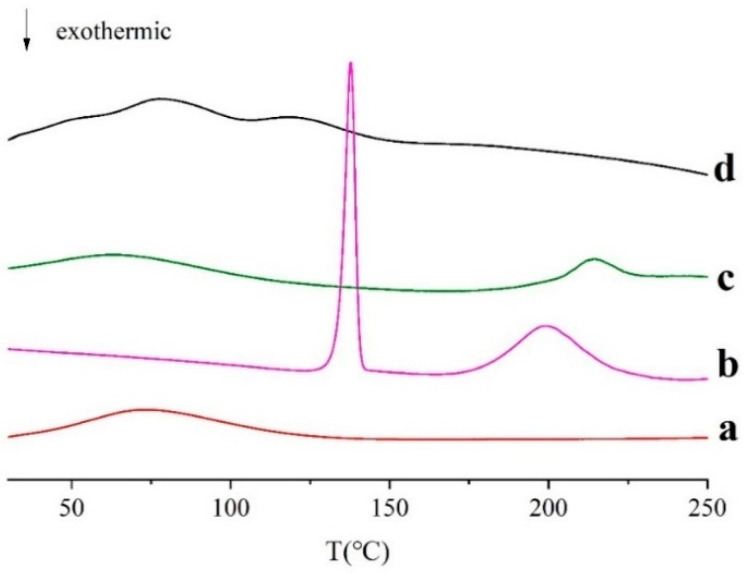
Differential scanning calorimetry (DSC) curves of (**a**) CS; (**b**) Man; (**c**) BSA, (**d**) Man-BSA-CS-NPs.

As shown in Figure 6, the unprocessed CS showed a wide peak of melting phase transition from 34 °C to 143 °C, and the peak is at 73 °C. This is because CS is an amorphous polymer mixture. For BSA, there is a large and broad endothermic peak between 32 and 164 °C, which is probably related to the evaporation of adsorbed water [41]. When heated to 200 °C, there is an endothermic peak in BSA, which represents thermal degradation of solid proteins. Man has a sharp melting endothermic peak at 138 °C and a wide endothermic peak with an extrapolated starting temperature (T_onset_) of 179 °C, and a peak temperature (T_peak_) of 221 °C. However, in Man-BSA-CS-NPs, the melting endothermic peak is transformed into a wide peak with multi-peak merging, and the characteristic peaks of Man and BSA disappeared, which indicates that CS, Man, and BSA interact with each other.

#### 2.3.3. Sodium Dodecyl Sulfate Polyacrylamide Gel Electrophoresis (SDS-PAGE) Analysis

**Figure 7:**
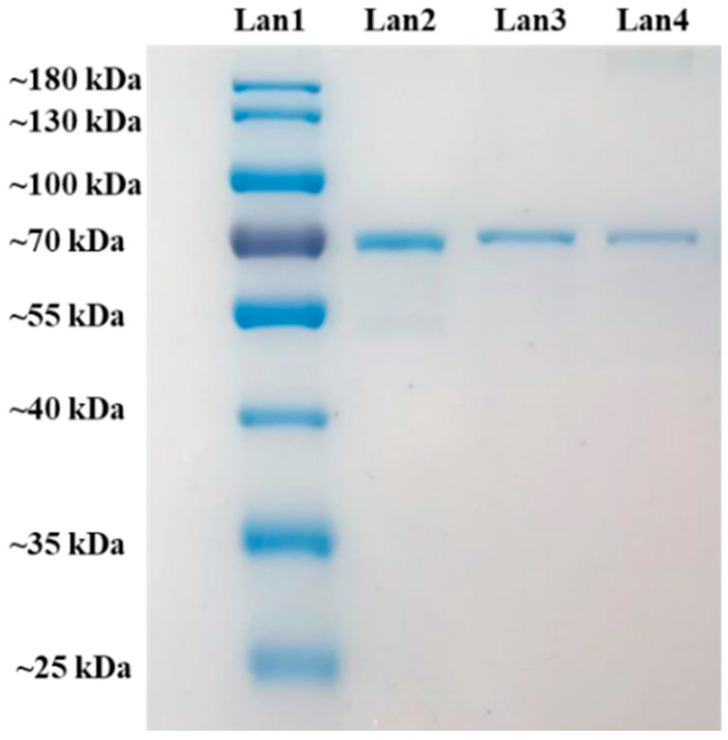
Sodium dodecyl sulfate polyacrylamide gel electrophoresis (SDS-PAGE) of MW ladder for comparison (Lan1), BSA reference (Lan2) and BSA from BSA-CS-NPs (Lan3) (CS concentration = 1 mg/mL, CS:TPP ratios (*w*/*w*) = 5:2.3, CS:TPP ratios (*v*/*v*) = 5:2) and Man-BSA-CS-NPs (Lan4) (CS concentration = 1 mg/mL, CS:TPP ratios (*w*/*w*) = 5:2.3, CS:TPP ratios (*v*/*v*) = 5:2, Man concentration = 3 mg/mL).

The integrity of BSA was investigated by SDS-PAGE. The molecular weight of BSA is 66.4 kDa. As shown in Figure 7, the bands of unprocessed BSA and BSA loaded in BSA-CS-NPs and Man-BSA-CS-NPs are almost in the same level, which indicates that there is no irreversible aggregation or degradation of BSA in BSA-CS-NPs and Man-BSA-CS-NPs.

### 2.4. Binding Parameters and Thermodynamic Analysis of CS-BSA and Man-CS-BSA Adducts by Fluorescence Spectroscopy

**Figure 8:**
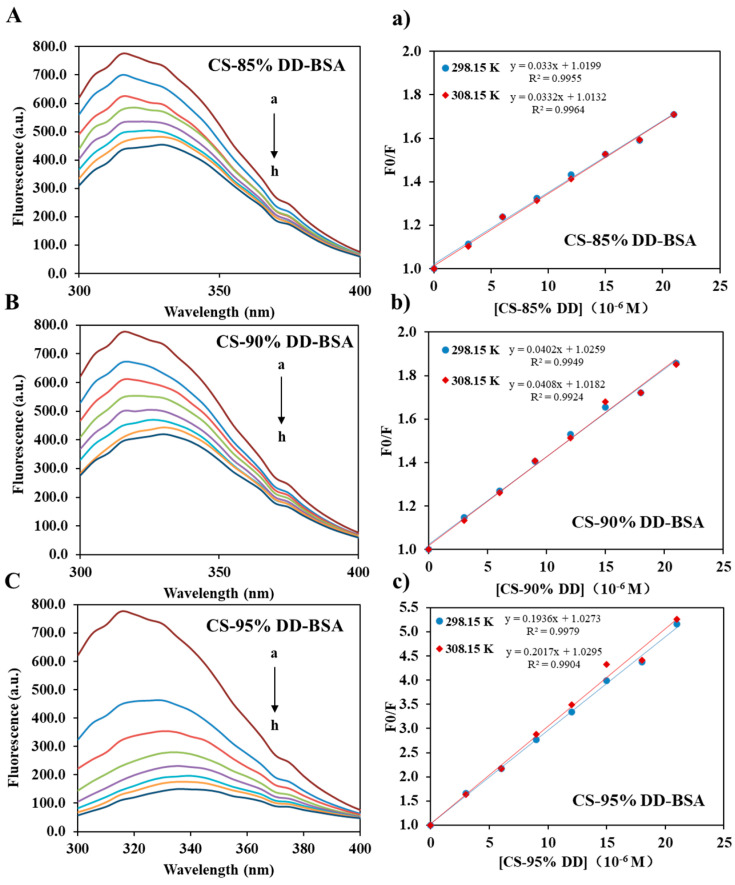
Steady-state fluorescence spectra of BSA in the absence and presence of CS (**A**–**C**) at 298.15 K, and Stern–Volmer plots (**a**–**c**) at 298.15 K and 308.15 K. [BSA] = 8 μM and [CS, a–h] = 0, 3, 6, 9, 12, 15, 18, 21 μM, λex = 280 nm.

Tryptophan fluorescence quenching is considered to be an effective method to investigate CS–protein interaction [36,42,43,44,45]. In our study, fluorescence quenching was used to investigate the binding affinity of CS and BSA. The quenching of tryptophan (Trp) may be caused by the collision process and/or the formation of complexes with the quenching agent. As shown in Figure 8, the fluorescence intensity of BSA decreased with the increasing CS concentration, which was due to the formation of CS-BSA adducts [46]. However, CS with different DD has different affinity to BSA, and the order of affinity was: 95% DD > 90% DD > 85% DD. The binding constant of CS-BSA complex was investigated by the Stern–Volmer equation (Equation (1)):

*F*_0_/*F* = 1 + *K*_SV_[*Q*] = 1 + *k*_q_*τ*_0_[*Q*] (1)

where *F*_0_ and *F* are the fluorescence intensity without and with quenching agent (CS solution), [*Q*] is the concentration of quenching agent, *K*_SV_ is the Stern–Volmer quenching constant, *k*_q_ is the quenching rate constant and *τ*_0_ is the lifetime of the fluorophore without quenching agent, which is 5.9 ns for BSA [47].

As shown in Table 3a, *K*_SV_ was obtained from the slope of the Stern–Volmer graph (Figure 8). *k*_q_ of CS-BSA adducts calculated by Equation (1) was greater than the limiting diffusion rate constant (2 × 10^10^ M^−1^s^−1^) [48]. It proves that the CS-BSA adducts were mainly quenched by a static quenching mechanism within the CS concentration range. However, the *K*_SV_ of all samples increased with the increasing temperature, which indicated that the quenching process was controlled by a hybrid mechanism of static and dynamic quenching. In the static quenching process, *K*_SV_ is regarded as the binding constant (*K*_Ch_) of the quencher and fluorophore [42]. It should be noted that the binding constants is correlated with the encapsulation and release of BSA. The fourth-order binding constants indicated a moderate affinity between CS and BSA [42]. As shown in Table 3a, the *K*_SV_ increased with the DD of CS. This indicates that the greater the DD extent of CS, the more stable the CS-BSA complex, and the encapsulation efficiency of BSA increased with the increasing DD extent of CS. These results are consistent with a previous report [36]. 

In order to elucidate the binding force between CS and BSA, the thermodynamic parameters of the complexes were calculated according to the van’t Hoff equation (Equation (2)) and the thermodynamic equations (Equations (3) and (4)). These formulas apply when ∆*H* does not change much within the temperature range studied. R is the universal gas constant (8.3145 J mol^−1^K^−1^).

ln(*K*_Ch(*T*2)_/K_Ch(*T*1)_) = ∆*H*(1/*T*_1_ − 1/*T*_2_)/*R* (2)

∆*G*(*T*) = −*RT* ln K_Ch(*T*)_ (3)

∆*S*(*T*) = −(∆*G* − ∆*H*)/*T* (4)

The thermodynamic parameters of CS-BSA are reported in Table 3b. All CS-BSA adducts had negative ∆*G* indicating that the binding of CS-BSA is spontaneous at the tested temperature. This is in line with those reported by previous research [42]. Moreover, all CS-BSA adducts have positive ∆*H* and ∆*S*, which indicates that hydrophobic bonds play a major role in CS–BSA interaction [42]. The hydrophobic groups of CS, such as -CH, -NH_2_ and -CH_3_ groups, may contributes to the hydrophobic bonds between CS and BSA. The -NH_2_ groups have a lower polarity than acetyl group. The increasing DD of CS means more -NH_2_ groups in CS, and thus, increasing hydrophobic bonds between CS and BSA. This explained that why CS with higher DD extent has stronger affinity with BSA. It is worth noting that the quenching of BSA by CS-NPs has both a static and a dynamic mechanism [47], which is similar to the interaction between CS and BSA. However, unlike the CS–BSA interaction (relies on hydrophobic bonds), the interaction between BSA and CS-NPs relies on van der Waals and hydrogen bonds [47].

**Figure 9:**
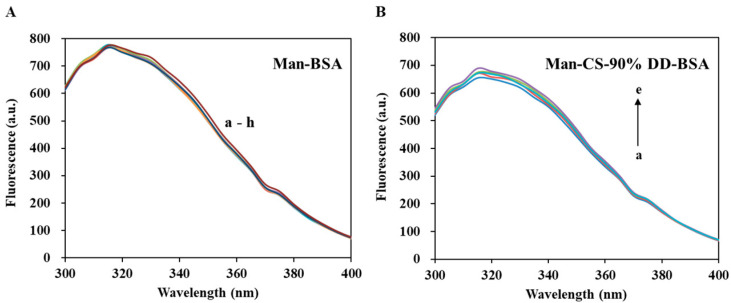
Fluorescence spectroscopy of Man-BSA and Man-CS-90%DD-BSA complexes: (**A**) Man-BSA, the concentrations of Man are with values of a–h (0, 1, 2, 3, 4, 5, 6 and 7 mg/mL); (**B**) Man-CS-90% DD-BSA, the concentrations of Man are with values of a–e (0, 1, 2, 3 and 4 mg/mL).

Figure 9 shows the interaction between Man and BSA. The results showed that Man has no significant quenching effect on BSA. The fluorescence intensity of CS-BSA complexes increased slightly with the increasing of Man concentration. This may be attributed to the formation of hydrogen bonds between Man and CS, or the -OH groups of Man occupying the -NH_2_ site of CS, hindering the binding of BSA and CS. 

In conclusion, the 90% DD CS we selected in this research, has an appropriate binding constant with BSA, which is required to balance the encapsulation and release of BSA in BSA-CS-NPs and Man-BSA-CDS-NPs. The formation of CS-BSA adducts can improve the stability of BSA. Furthermore, the Man we added into Man-BSA-CDS-NPs decreased the binding constant between CS and BSA slightly, which may reduce of BSA encapsulation efficiency.

### 2.5. Cytotoxicity and Cell Uptake Study

**Figure 10:**
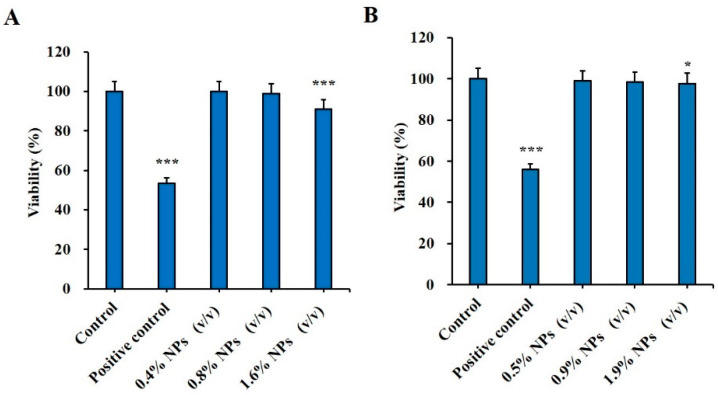
Cytotoxic effect of Man-BSA-CS-NPs on (**A**) HNEpC cells at a concentration of 0.4–1.6% (*v*/*v*) and on (**B**) DC2.4 at a concentration of 0.5–1.9% (*v*/*v*); 15% (*v*/*v*) dimethyl sulfoxide (DMSO) were taken as a positive control; cells that did not undergo any treatment were used as a negative control. Relative cell viability levels were determined by MTT assay (mean ± SD, *n* = 3, * *p* < 0.05, *** *p* < 0.001 compared with the control).

Analysis of the cytotoxicity of the vaccine carrier is crucial to confirm the safety of NPs. In this study, the cytotoxicity effect of Man-BSA-CS-NPs was assessed via a MTT assay. The relative viability of HNEpC and DC2.4 cells at 6 h post exposure can be seen in Figure 10. Man-BSA-CS-NPs induced almost no changes in HNEpC and DC2.4 cells, as the viability declined only to 91% (Figure 10A) and 97.7% (Figure 10B) in the worst case. The results indicated good biocompatibility and low toxicity of Man-BSA-CS-NPs. Statistically significant differences occurred above 1% (*v*/*v*) concentration. Nevertheless, higher concentrations of Man-BSA-CS-NPs only slightly increased the cytotoxicity.

**Figure 11:**
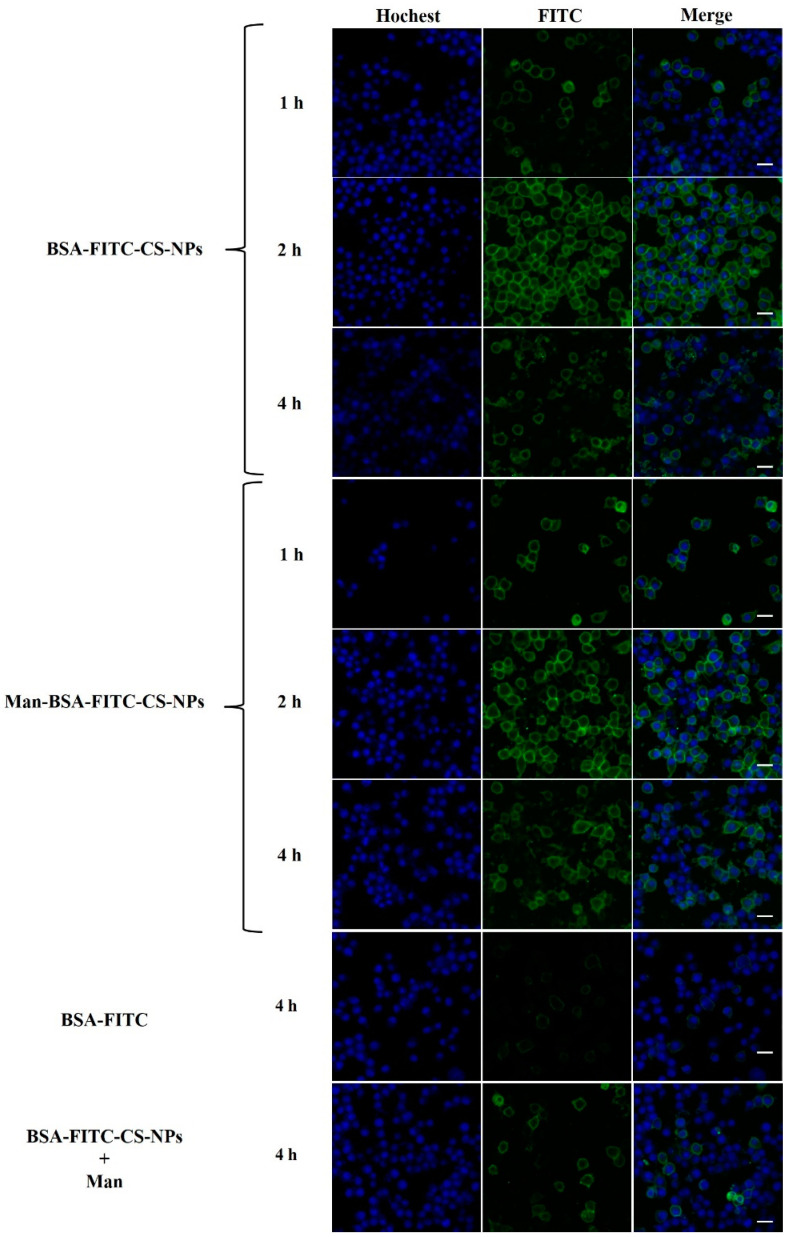
The cellular uptake of different formulations in DC2.4 cells at different time (1 h, 2 h, 4 h) observed by confocal microscope. Scale bar: 20 µm.

CS is a strong polycation with high charge density and readily binds to negatively charged cell membranes and increases endocytosis of biological macromolecules in the cells. The results in Figure 11 demonstrate that BSA-FITC was endocytosed at much higher efficiency with CS-NPs/Man-CS-NPs compared to the control group with BSA-FITC alone. There is a tendency that Man-modified BSA-FITC-CS-NPs speed up and promote the process of cell uptake and internalization of BSA-FITC in DC2.4 cells. However, the addition of Man to BSA-FITC-CS-NPs suspension had an inhibitory effect on endocytosis and internalization. This may be because Man occupies MR and acts as a competitive inhibitor of MR.

### 2.6. In Vivo Immunogenicity

**Figure 12:**
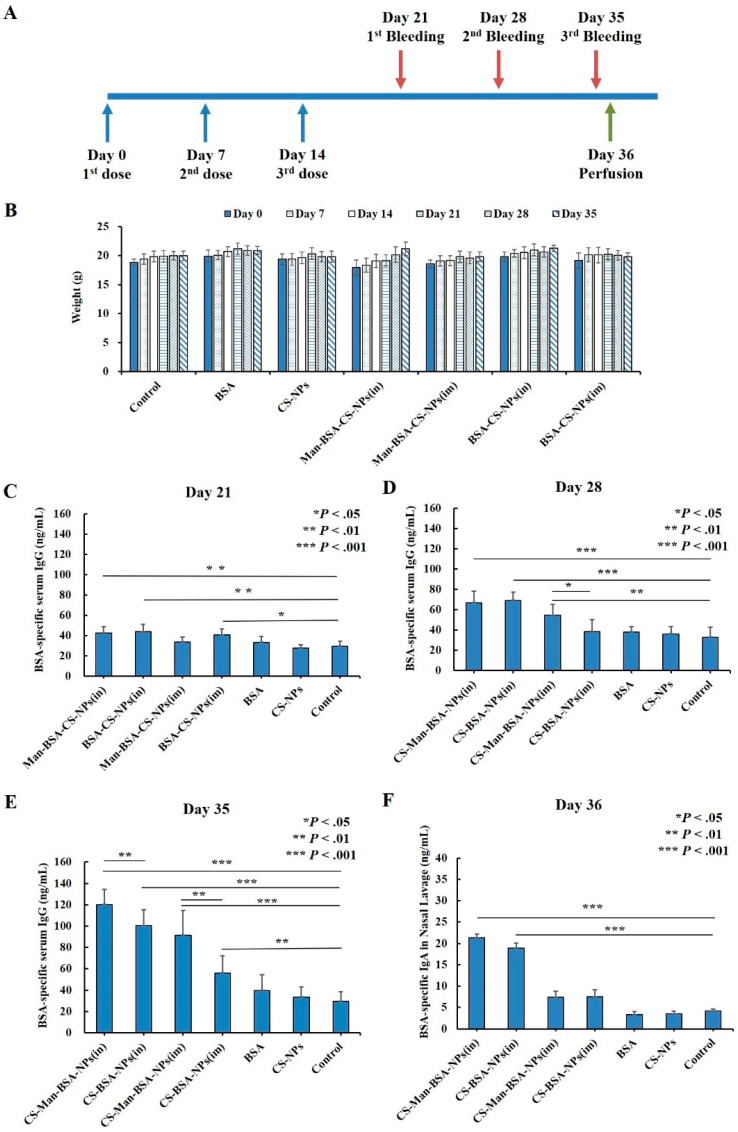
Immunization and sampling timeline (**A**); changes in body weight of mice (**B**); BSA-specific serum IgG (**C**–**E**) and nasal lavage fluid (NLF) IgA (**F**) antibody responses; all results are reported as mean (SD) (*n* = 6, * *p* < 0.05, ** *p* < 0.01, *** *p* < 0.001).

As shown in Figure 12A, to evaluate the immunogenicity of Man-BSA-CS-NPs upon mucosal administration, mice were intranasally (IN) immunized with unprocessed BSA solution, CS-NPs, BSA-CS-NPs, or Man-BSA-Cs-NPs three times on Days 0, 7, and 14. In addition, two groups of mice were IM immunized with BSA-CS-NPs or Man-BSA-CS-NPs as a control to IN administration. The mice in the control group did not receive any treatment and served as a negative control. The results in Figure 12B showed no adverse reactions of abnormal weight loss occurred in all groups of mice during the whole experimental process. 

Humoral immune response was determined by analysis of BSA-specific IgG antibody levels in the serum. Mice immunized with Man-BSA-CS-NPs (in) and BSA-CS-NPs (in) showed significantly higher BSA-specific serum IgG antibody responses compared to responses of the control group from Days 21 to 35 (Day 21: *p* < 0.01; Day 28,35: *p* < 0.001) (Figure 12C–E)). On Day 35 (Figure 12E), the BSA-specific serum IgG antibody induced by Man-BSA-CS-NPs (in) had significantly higher responses compared to BSA-CS-NPs (in) (*p* < 0.01). The BSA-specific IgG expression levels induced by BSA or CS-NPs were not significantly higher than those in the control group from the beginning to the end. 

As for IM immunized groups, mice immunized with BSA-CS-NPs (im) showed significantly higher BSA-specific serum IgG antibody responses (Figure 12C,E)) compared to responses of the control group on Day 21 (*p* < 0.05) and Day 35 (*p* < 0.01). However, it is worth noting that the significant difference disappeared on Day 28, indicating that the difference on Day 21 may be a weak false positive. On Days 28 and 35 (Figure 12D,E)), the BSA-specific serum IgG antibody induced by Man-BSA-CS-NPs (im) had significantly higher responses compared to BSA-CS-NPs (im) (Day 28: *p* < 0.05; Day 35: *p* < 0.01).

The results for Day 35 (Figure 12E) showed the highest level of BSA-specific serum IgG expression. The results showed that BSA-CS-NPs induced increased BSA-specific serum IgG antibody responses and additional modification of Man into the BSA-CS-NPs further enhanced IgG antibody responses. Compared to corresponding IM immunization, IN immunization induced an increased BSA-specific serum IgG antibody response (BSA-CS-NPs (in-im): *p* < 0.001; Man-BSA-CS-NPs (in-im): *p* < 0.05).

In order to analyze the mucosal immune responses, BSA-specific IgA antibodies in NLF were determined (Figure 12E). The results showed that the response of BSA-specific IgA was increased significantly with Man-BSA-CS-NPs and BSA-CS-NPs immunization by the IN route in contrast to IM injection (Man-BSA-CS-NPs: *p* < 0.01; BSA-CS-NPs: *p* < 0.05) and Control group (*p* < 0.001). However, there is no significant difference between the BSA-specific IgA response induced by Man-BSA-CS-NPs (in) and BSA-CS-NPs (in). 

## 3. Materials and Methods

### 3.1. Materials

Chitosan (85% DD, 90% DD, 95% DD, density of CS is higher than 0.6 g/mL) and sodium tripolyphosphate (TPP) were purchased from Macklin (Shanghai, China). D-mannose (Man) were purchased from Solarbio (Beijing, China). Bovine serum albumin (BSA) was purchased from Macgene (Beijing, China). SDS-PAGE Gel Preparation Kit and protein loading buffer (5×) were purchased from Beyotime Biotechnology (Shanghai, China). MTT Cell Proliferation and Cytotoxicity Assay Kits, BSA-FITC, Hoechst 33,258 and Coomassie blue were purchased from Solarbio (Beijing, China). All other reagents used in the study were purchased from Sinopharm Chemical Reagent (Shanghai, China) with analytical grade.

### 3.2. Preparation of Chitosan Nanoparticles (CS-NPs)

CS-NPs were prepared by the ionic gelation method [49], using 90% DD CS. 90% DD CS was dissolved in 0.15% acetic acid solution to prepare CS solution (pH 4.0), and TPP was dissolved in MilliQ water to prepare the TPP solution. The volume ratio of CS solution: TPP solution was kept at 5:2 for all the preparation experiments. Briefly, the TPP solution was slowly added into the CS solution under magnetic stirring at room temperature, and CS-NPs spontaneously formed. After centrifugation (15,000 rpm, 30 min), the sediment was dispersed in 2 mL deionized (DI) water. The corresponding preparation conditions of each numbered sample are shown in Table 4. 

BSA-loaded CS-NPs (BSA-CS-NPs) were prepared by dissolving BSA into the CS solution, and then, we carried out the same preparation procedures with the CS-NPs. Similarly, Man-modified BSA-CS-NPs (Man-BSA-CS-NPs) were obtained by dissolving Man and BSA into the CS solution, followed by the same mixing method.

### 3.3. Design of Experiment (DoE)

Minitab 10 software was used for DoE, which was based on a full-factor test. We conducted 27 trials with three variables (factors) at three levels, using a 3^3^ full-factor design. Response surface methodology (RSM) is a powerful data modelling tool [50]. In this study, RSM was used for representing complex nonlinear relationships between independent variables and responses. After optimization, three additional experiments under optimized condition were conducted to compare with the predicted results. 

For Man-BSA-CS-NPs, the variables and their three levels are shown in Table 5a. The concentration of CS solution was kept at 1.0 mg/mL while TPP was 1.15 mg/mL. The composition of 27 formulations and the mean values of responses are shown in Table 5b.

### 3.4. Characterization of NPs

#### 3.4.1. Particle Size, Zeta Potential and Polydispersity index (PDI)

We diluted the prepared suspension of NPs to an appropriate concentration. The particle size, zeta potential, and polydispersity index (PDI) of NPs were determined by Zetasizer Nano ZS90 (Malvern, UK) at room temperature through dynamic light scattering (DLS). Three analyses were performed for each sample.

#### 3.4.2. Transmission Electron Microscope (TEM)

The morphology of nanoparticle was observed by transmission electron microscopy (TEM) (H-7650, Hitachi, Tokyo, Japan). The nanoparticle suspension was diluted with MilliQ water, and then dropped onto a copper grid. After being air-dried at room temperature, the sample was installed on the instrument and observed and photographed.

#### 3.4.3. Fourier Transform Infrared Spectroscopy (FTIR) 

Lyophilized CS-NPs or ingredients were mixed with KBr powder and pressed into flakes. Fourier transform infrared spectroscopy (FTIR) was collected by Bruker Tensor 27 (Bruker Optics, Billerica, MA, USA). All samples were measured in the frequency range of 500–4000 cm^−1^ with a resolution of 3.814 cm^−1^. A total of 32 scans were carried out and the signals were averaged.

#### 3.4.4. Differential Scanning Calorimetry (DSC) 

Differential scanning calorimetry (DSC) was performed by a DSC 200F3 (Netzsch, Germany), which was equipped with automatic injector, cooling unit and advanced analysis software. The flow rate of nitrogen gas was 20 mL/min to provide an inert atmosphere and prevent oxidation during the measurement. Samples of about 2 mg were placed in an aluminum crucible and covered with holes. These samples were heated from 25 °C to 300 °C at a rate of 5 °C/min for DSC analysis.

#### 3.4.5. Sodium Dodecyl Sulfate Polyacrylamide Gel Electrophoresis (SDS-PAGE)

BSA-CS-NPs or Man-BSA-CS-NPs were dispersed into PBS with the concentration of 5 mg/mL BSA equivalent for 12 h incubation at 36 °C. After incubation, the dispersion was centrifuged and the supernatant was collected. Subsequently, the supernatant and 1 mg/mL standard BSA solution were boiled for 5 min in the protein loading buffer containing 5% mercaptoethanol and 2.5% SDS. The SDS-PAGE experiment then followed the previously reported standard operating procedure [51] by using a gel composed of 5% concentrated gel and 10% separated gel. The gel was photographed after Coomassie blue staining and dye-decolorizing.

### 3.5. Investigation of the Interaction between Bovine Serum Albumin (BSA) and CS, and between BSA and Man by Fluorescence Spectroscopy

The tryptophan (Trp) fluorescence of BSA was evaluated by fluorescence spectroscopy. CS and Man were dissolved in Tris-HCl aqueous solution (0.5 mM, pH 5–6) to prepare CS solution (85% DD, 90% DD and 95% DD, 3, 6, 9, 12, 15, 18, 21 μM) and Man solution (1, 2, 3, 4, 5, 6, 7 mg/mL). BSA was dissolved into CS solution or Man solution with the concentration of 8 μM. To investigate the effect of Man on CS-BSA adducts, Man was added to the 90% DD CS (3 μM)-BSA (8 μM) solution with the concentration of 1, 2, 3, 4, 5, 6, 7 mg/mL, respectively. The excitation wavelength was set at 285 nm and the emission spectra was monitored from 300 nm to 500 nm with a step-width of 5 nm. Measurements were made at 298.15 and 308.15 K. The quenching parameters were calculated based on intensity formed by the fluorescence peak, which was the mean value of three repeated trials.

### 3.6. Cell Study

#### 3.6.1. Cell Culture

Dendritic cells (DC2.4 cell line) and human nasal epithelial cells (HNEpC) were purchased from the China Center for Type Culture Collection (CCTCC). DC2.4 cells were cultured on dishes in complete medium containing DMEM, 10% fetal bovine serum and 1% of penicillin-streptomycin solution while HNEpC were cultured with MEM, 10% fetal bovine serum and 1% of penicillin-streptomycin solution. Cells were incubated in thermotank with 5% CO_2_ at 37 °C. Cells were passaged using standard cell culture techniques. The medium of DC2.4 cells was refreshed at intervals of 2 days. The medium of DC2.4 cells was updated every 2 days, while the medium of HNEpC was refreshed at intervals of 3 days.

#### 3.6.2. Cytotoxicity Testing with MTT Method

MTT assay (3-(4,5-dimethyl-2-thiazolyl)-2,5-diphenyl-2-H-tetrazolium bromide, MTT) was used to study the cytotoxicity of Man-BSA-CS-NPs in DC2.4 cells and HNEpC. The initial suspension of Man-BSA-CS-NPs was centrifuged from 30 mL to 1 mL by an ultrafiltration centrifuge (100 Kda, 1000 RPM). Then, the suspension was diluted in the complete medium of DC2.4 cells/HNEpC, respectively. 

DC2.4 cells/HNEpC were seeded in 96-well plates with 200 mL medium/well at a density of 1 × 10^4^ cells/well for 24 h. After discarding the medium and washing cells with PBS, DC2.4 cells/HNEpC were treated with serial concentrations of Man-BSA-CS-NPs (200 μL to each well) and incubated for 6 h. Cells with only complete medium were used as a control, while cells treated with 15% dimethyl sulfoxide (DMSO) (*v*/*v*) in culture medium were taken as a positive control. Other operations followed the instructions of MTT kit (Solarbio, Beijing, China). We mixed 10 μL MTT solution with 90 μL culture medium added into each well, followed by further incubation in the dark at 37 °C for 4 h. We added 110 μL DMSO to each well after discarding the culture medium. The plates were incubated at room temperature on a shaker at low speed (100 RPM) for 10 min. The optical density (OD) was measured by a microplate reader (Thermo, Varioskan lux, Waltham, MA, USA) at 490 nm. The results are expressed as the relative cell viability (%) in comparison to untreated controls. All of the cytotoxicity assay experiments were performed in more than octuplicate.

#### 3.6.3. Cell Uptake Study

Purified BSA-FITC was used to replace BSA to prepare BSA-FITC-CS-NPs and Man-BSA-FITC-CS-NPs under the optimized prescription and preparation conditions. The initial suspension of NPs was centrifuged from 30 mL to 1 mL by ultrafiltration centrifuge (100 Kda, 1000 RPM), and then suspended into the culture medium. The final concentration of each NPs was 2% (*v*/*v*).

Cellular uptake in the DC2.4 cells was examined by confocal laser scanning microscopy. The DC2.4 cells were seeded in Confocal dish (1 × 10^5^ cells/dish) and incubated for 24 h at 37 °C. with 5% CO_2_. 1 mL of 2% (*v*/*v*) BSA-FITC-CS-NPs or Man-BSA-FITC-CS-NPs suspension (containing 200 μg of BSA-FITC) was added to each dish and co-cultured with the DC2.4 cells for 1 h, 2 h and 4 h, respectively. BSA-FITC (2 µg/µL) was used as negative control, co-cultured with the cells for 4 h. DC2.4 cells co-cultured with 2% (*v*/*v*) BSA-FITC-CS-NPs and Man (2 µg/µL) for 4 h were taken as another control. After treatment, cells were washed twice with PBS. Nuclei were observed by Hoechst 33,258 staining. The cells were incubated with Hoechst 33,258 (10 µg/mL × 2 mL/well) at room temperature for 5 min and were then washed by PBS thrice. Finally, the cell medium was replaced with HBSS and stored at 4 °C under dark conditions until detection. Fluorescence intensity was measured with an upright laser scanning confocal microscope with multiphoton (NLO) laser (Carl Zeiss, LSM880, Oberkohen, Germany) using its analysis program. All of the cell uptake experiments were performed in triplicate.

### 3.7. Animal Study

#### 3.7.1. Animal Culture

Female BALB/c mice (6–8 weeks of age) were purchased from Beijing Vital River Laboratory Animal Technology Co., Ltd., (Beijing, China). All the animal experiments were performed in accordance with the Experimental Animal Administrative Committee of Beijing Institute of Pharmacology and Toxicology.

#### 3.7.2. Intranasal (IN) Administration and Intramuscular (IM) Injection

The corresponding NPs suspensions were performed as described above. The mice were divided into seven groups of six mice. IN administration was performed according to a procedure modified from the method of Capsoni [52]. For one single IN injection, mice were administered either with BSA solution (1 µg/µL, 10 µL/naris, Total 20 µL) (Group BSA), CS-NPs suspension (10 µL/naris, Total 20 µL) (Group CS-NPs), BSA-CS-NPs suspension (10 µL/naris, Total 20 µL) (Group BSA-CS-NPs(in)) or Man-BSA-CS-NPs suspension (10 µL/naris, Total 20 µL) (Group Man-BSA-CS-NPs(in)). For one single IM injection, Mice were injected with 20 µL BSA-CS-NPs suspension (Group BSA-CS-NPs(im)) or 20 µL Man-BSA-CS-NPs suspension (Group Man-BSA-CS-NPs(im)) into the left caudal thigh muscles. Mice in each group were given IN/IM administration on Day 0, Day 7 and Day 14, thrice in total. Mice in the control group did not receive any treatment.

#### 3.7.3. Antibody Concentrations of Serum and Nasal Lavage Fluid (NLF)

Blood samples were collected via retinal venous plexus on the Day 21, Day 28 and Day 35. After coagulating at 4 °C, blood samples were centrifuged at 5000 rpm, 4 °C for 15 min. Then serum was collected and stored at −80 °C. NLF was collected on the day after the last bleeding (Day 36). To obtain NLF samples, a previously described trans-pharyngeal technique [53] was used. The volume of 1 mL PBS was instilled, and the fluid was collected from the nostril. We repeated the process with the collected fluid. After five repetitions, the NLF was centrifuged (5000 rpm, 4 °C, 15 min), and the isolated supernatant was stored at −80 °C until assayed.

The concentrations of anti-BSA IgG in serum and anti-BSA IgA in NLF were measured using sensitive sandwich ELISA kits (Adanti Biotechnology Co., Ltd., Wuhan, China), in accordance with the manufacturer’s instructions.

### 3.8. Statistical Analysis

In this study, SPSS13.0 software was used to perform statistical analysis. The data were tested by analysis of variance (ANOVA). *p* < 0.05 was considered statistically significant. 

## 4. Conclusions

In this study, we prepared BSA-loaded CS-NPs with Man modification by an ionic gelation method, which is environmentally friendly and simple. DoE was used to optimize the particle size, PDI and zeta potential of our products. 

Man has no significant influence on the size/PDI/zeta potential of Man-BSA-CS-NPs, which indicates a large prescription adjustment range. The optimized Man-BSA-CS-NPs were near-spherical with uniform density and dispersion. The Man-BSA-CS-NPs were characterized by TEM, FTIR, DSC and SDS-PAGE analyses. FTIR and DSC results confirmed the presence of Man in Man-BSA-CS-NPs. In addition, the BSA released from Man-BSA-CS-NPs has no irreversible aggregation or degradation.

The cell viability tests demonstrated a lack of cytotoxicity for Man-BSA-CS-NPs. For in vivo studies in mice, there was no adverse reaction of abnormal weight loss after IN/IM was immunized with Man-BSA-CS-NPs. All these results indicated good biocompatibility and low toxicity of Man-BSA-CS-NPs. Man-modified BSA-FITC-CS-NPs speed up and promote the process of cell uptake and internalization of BSA-FITC in DC2.4 cells. It is also worth mentioning that the same dose of Man-BSA-CS-NPs showed the strongest immune response after IN immunization of mice, which indicates that our study provides a promising method to modify CS-NPs for intranasal vaccine delivery and Man-CS-NPs can be further developed as a promising vaccine delivery system. 

## Figures and Tables

**Table 1:** Dynamic light scattering (DLS) detection of CS-NPs prepared by using different CS/TPP mass ratios.

Lot#	CS:TPP (*w*/*w*)	Particle Size ± SD (nm)	PDI	Zeta (mV)
CS-NP-2-1	2.2:1	246.1 ± 12.7	0.493 ± 0.043	36.4 ± 0.4
CS-NP-2-2	2.3:1	194.0 ± 3.6	0.325 ± 0.028	37.3 ± 0.1
CS-NP-2-3	2.4:1	217.5 ± 3.1	0.384 ± 0.011	40.2 ± 1.1

The concentration of CS solution was 1 mg/mL; CS solution was kept at 5 mL with pH 4.0; the volume ratio of CS solution: TPP solution was kept at 5:2 for all the preparation experiments; all results are reported as mean (SD), *n* = 3.

**Table 2:** Calculated optimization of Man-BSA-CS-NPs by the Minitab 10 software.

Value	Mannose (Man) (3 mg/mL)	Co-Mixing Time (24 h)	Bovine Serum Albumin (BSA) (250 μg)
	Optimization Responses	Experimental Results	
Particle size	156 ± 10 nm	142 ± 3 nm	
PDI	0.206 ± 0.021	0.183 ± 0.019	
Zeta potential	33.5 ± 1.2 mV	32.9 ± 0.4 mV	

**Table 3:** (a) The quenching constants of BSA by CS (85% DD, 90% DD and 95% DD) at two different temperatures. (b) Thermodynamic parameters of CS (85% DD, 90% DD and 95% DD) forming adducts with BSA.

(**a**)						
**CS-BSA Complexes**				**Quenching Constants**		
				***T* (K)**	***K*_SV_ (M^−1^)**	***K*q (M^−1^s^−1^)**
CS-85% DD-BSA				298	3.30 × 10^4^	5.59 × 10^12^
				308	3.32 × 10^4^	5.63 × 10^12^
CS-90% DD-BSA				298	4.02 × 10^4^	6.81 × 10^12^
				308	4.08 × 10^4^	6.92 × 10^12^
CS-95% DD-BSA				298	1.94 × 10^5^	3.28 × 10^13^
				308	2.02 × 10^5^	3.42 × 10^13^
(**b**)						
**Chitosan-Protein Complexes**	***T* (K)**	**∆*H* (kJ mol^−1^)**	**∆*S* (mol^−1^ K^−1^)**		**∆*G* (kJ mol^−1^)**	**Nature of the Binding Forces**
CS-85% DD-BSA	298	0.69	88.05		−25.78	∆*H* > 0 and ∆*S* > 0, Hydrophobic forces
	308		88.05		−26.66	
CS-90% DD-BSA	298	1.13	91.94		−26.27	∆*H* > 0 and ∆*S* > 0, Hydrophobic forces
	308		91.94		−27.19	
CS-95% DD-BSA	298	3.13	111.71		−30.16	∆*H* > 0 and ∆*S* > 0, Hydrophobic forces
	308		111.71		−31.28	

**Table 4:** Preparation conditions of chitosan nanoparticles (CS-NPs) samples.

Lot#	CS (mg/mL)	TPP (mg/mL)	CS:TPP (*w*/*w*)	CS:TPP (*v*/*v*)
CS-NP-1-1	1	2	1.25:1	5:2
CS-NP-1-2	1	1	2.5:1	5:2
CS-NP-1-3	1	0.5	5:1	5:2
CS-NP-2-1	1	1.15	2.2:1	5:2
CS-NP-2-2	1	1.1	2.3:1	5:2
CS-NP-2-3	1	1.05	2.4:1	5:2

**Table 5:** The 2^3^ full factorial design of experimental sites for Man-BSA-CS-NPs (*n* = 3). (a) Values of levels of process parameters. (b) Experimental design and experimental values of particle size, PDI and zeta potential.

(**a**)						
**Variable Factor**				**Low Level**	**Mid-Level**	**Low Level**
Concentration of Mannose (Man) X1 (mg/mL)				1	2	3
The blending time of CS and Mannose (Co-Mixing Time) X2 (h)				6	12	24
Amount of bovine serum albumin (BSA) X3 (μg)				250	500	1000
(**b**)						
**Run**	**Man** **(mg/mL)**	**Mixing Time** **(h)**	**BSA** **(μg)**	**Particle Size** **(nm)**	**PDI**	**Zeta** **(mV)**
1	1	6	250	189.1 (±2.3)	0.324 (±0.044)	33.8 (±0.5)
2	1	6	500	176.9 (±1.0)	0.287 (±0.015)	33.6 (±0.9)
3	1	6	1000	192.4 (±2.2)	0.272 (±0.008)	37.7 (±0.7)
4	1	12	250	155.1 (±1.3)	0.218 (±0.017)	32.9 (±0.8)
5	1	12	500	150.4 (±3.0)	0.200 (±0.011)	30.8 (±1.1)
6	1	12	1000	141.8 (±3.0)	0.211 (±0.005)	31.6 (±1.1)
7	1	24	250	183.3 (±0.9)	0.170 (±0.007)	29.0 (±0.7)
8	1	24	500	172.8 (±2.3)	0.223 (±0.009)	34.7 (±1.0)
9	1	24	1000	189.9 (±2.4)	0.260 (±0.013)	34.3 (±1.0)
10	2	6	250	161.6 (±4.1)	0.211 (±0.016)	32.9 (±0.5)
11	2	6	500	164.3 (±4.9)	0.200 (±0.015)	31.4 (±0.8)
12	2	6	1000	172.7 (±3.7)	0.197 (±0.012)	30.4 (±0.5)
13	2	12	250	161.7 (±5.4)	0.246 (±0.004)	34.2 (±0.3)
14	2	12	500	161.8 (±2.6)	0.234 (±0.016)	33.6 (±0.4)
15	2	12	1000	169.7 (±4.3)	0.258 (±0.008)	30.9 (±0.6)
16	2	24	250	179.0 (±2.6)	0.224 (±0.019)	32.7 (±0.4)
17	2	24	500	141.3 (±3.6)	0.190 (±0.024)	32.9 (±0.4)
18	2	24	1000	158.4 (±4.2)	0.189 (±0.009)	36.4 (±1.5)
19	3	6	250	153.3 (±4.3)	0.211 (±0.026)	31.7 (±0.9)
20	3	6	500	172.1 (±5.3)	0.218 (±0.004)	30.2 (±1.5)
21	3	6	1000	161.4 (±4.0)	0.228 (±0.017)	30.7 (±1.2)
22	3	12	250	161.8 (±2.5)	0.224 (±0.018)	33.7 (±0.4)
23	3	12	500	150.9 (±5.2)	0.233 (±0.015)	32.6 (±0.9)
24	3	12	1000	160.6 (±2.7)	0.232 (±0.003)	32.9 (±0.6)
25	3	24	250	142.3 (±2.9)	0.183 (±0.019)	32.9 (±0.4)
26	3	24	500	177.0 (±5.2)	0.248 (±0.013)	33.3 (±0.6)
27	3	24	1000	170.1 (±3.7)	0.240 (±0.019)	31.6 (±0.7)

## Data Availability

Not applicable.

## Sample Availability

Samples of the compounds are not available from authors.
